# An Empty Scoping Review on the Financing of Necrotizing Periodontal Disease Prevention and Control in Africa: Implications for Global Oral Health Research, Policy, and Practice

**DOI:** 10.1002/hsr2.71750

**Published:** 2026-02-11

**Authors:** Kafayat Aminu, Yovanthi Anurangi Jayasinghe, Oluwatobi Emmanuel Adegbile, Adetayo Olorunlana, Rita Amarachi Nwebo, Precious Chika Nnannah, Afeez Abolarinwa Salami, Akinyele Olumuyiwa Adisa, Ugochukwu Anthony Eze, Kehinde Kazeem Kanmodi

**Affiliations:** ^1^ Centre for Evidence Synthesis and Implementation Research, Cephas Health Research Initiative Inc. Ibadan Nigeria; ^2^ Centre for Disease Control and Prevention, Cephas Health Research Initiative Inc. Ibadan Nigeria; ^3^ Scientific Advisory Board, Cephas Health Research Initiative Inc. Ibadan Nigeria; ^4^ College of Health Sciences Caleb University Imota Nigeria; ^5^ Centre for Digital Health Research, Innovation and Practice, Cephas Health Research Initiative Inc. Ibadan Nigeria; ^6^ Centre for Dental and Craniofacial Research, Cephas Health Research Initiative Inc. Ibadan Nigeria; ^7^ Office of the Executive Director, Cephas Health Research Initiative Inc. Ibadan Nigeria; ^8^ Department of Oral Pathology and Oral Medicine University of Ibadan Ibadan Nigeria; ^9^ School of Health and Life Sciences Teesside University Middlesbrough UK; ^10^ School of Public Health Thomas Adewumi University Oko Nigeria

**Keywords:** Africa, control, funding sources, health policy, necrotizing periodontal disease, oral health financing, prevention, review

## Abstract

**Background and Aims:**

Necrotizing periodontal diseases (NPDs) represent a significant yet neglected burden in sub‐Saharan Africa, often exacerbated by poverty, malnutrition, HIV, and limited access to oral healthcare. Despite their public health relevance, financing mechanisms supporting their prevention and control in African settings are limited. This scoping review aimed to identify and map existing literature on the financing of NPD prevention and control in Africa and to highlight implications for global oral health policy, research, and practice.

**Methods:**

This scoping review adhered to the step‐by‐step approach of Arksey and O'Malley and the PRISMA‐ScR guidelines. A comprehensive search was conducted across several databases, including PubMed, SCOPUS, AMED, CINAHL Ultimate, Dentistry and Oral Sciences Source, SPORTDiscus with Full Text, APA PsycArticles, Psychology and Behavioral Sciences Collection, Regional Business News, and APA PsycInfo. Keywords related to the financing of NPD prevention and control in Africa were used. Articles were imported into Rayyan for deduplication and screened through title and abstract review based on predefined inclusion and exclusion criteria.

**Results:**

A total of 63 studies were identified, and 25 duplicate records were removed on Rayyan. Following the deduplication process, the remaining 38 studies were subjected to title/abstract screening, and no study was selected for full‐text review or subsequent analysis since the inclusion requirements were not fulfilled.

**Conclusion:**

This empty scoping review provides valuable insights into the current state of global oral health financing by highlighting a critical gap in the literature related to the prevention and control of NPDs in Africa. The absence of eligible studies underscores the lack of focused research and policy attention on financing strategies for NPD‐related interventions in low‐resource settings. This gap presents a missed opportunity to inform effective, context‐specific, and sustainable approaches to managing a neglected yet burdensome group of oral diseases.

## 1 Introduction

Periodontal diseases are prevalent oral health conditions that impact a significant portion of the global population [1, 2, 3]. Approximately 3.68 billion people globally suffer from severe periodontitis, with two in five adults affected in the United States [2, 4, 5, 6]. Although comprehensive data for specific African countries are still emerging, the Global Burden of Disease Study 2021 indicated a reduction in the prevalence of severe periodontitis in Sub‐Saharan Africa, declining from an estimated 19,577 cases per 100,000 individuals in 2019 (95% uncertainty interval: 18,272–20,954) to 12,000 cases per 100,000 individuals in 2021 (95% uncertainty interval: 9980–14,000) [4, 7]. Despite this improvement, the burden remains elevated due to socioeconomic disparities, limited access to oral healthcare, and a potentially higher prevalence of contributing factors such as malnutrition, HIV, and other systemic conditions [8, 9, 10, 11]. Necrotizing periodontal diseases (NPDs), including necrotizing gingivitis, necrotizing periodontitis, and necrotizing stomatitis, present a unique and severe challenge, particularly among vulnerable populations in Africa [12, 13, 14, 15]. The pathogenesis of NPDs is multifactorial, involving the interplay of bacterial biofilms, host response, and various predisposing factors [16, 17]. Moreover, NPDs are often associated with acute infections and are exacerbated by factors such as malnutrition, systemic diseases, and poor access to healthcare, creating a distinct clinical entity [18, 19, 20].

Oral health financing in Africa faces significant challenges, including insufficient national budget allocation, reliance on out‐of‐pocket payments, and the exclusion of oral health from universal health coverage frameworks [21, 22, 23]. Evidence suggests that only 0.1%–0.5% of the gross domestic product (GDP) in African countries is allocated to science and technology, with even less directed toward dental research and oral health interventions [13, 24, 25]. This underinvestment worsens access inequities, particularly in rural areas where oral health professionals are scarce, and facilities lack resources [24]. The burden of NPDs is further compounded by the absence of targeted public health interventions, despite evidence supporting the effectiveness of prevention, early diagnosis, and treatment strategies [26, 27].

Innovative strategies, such as task‐shifting to mid‐level workers, deploying mobile dental units, and integrating oral health into broader non‐communicable disease (NCD) prevention efforts, have shown promise in enhancing accessibility and cost‐efficiency [13, 28]. Mobile dental clinics in South Africa achieved a cost‐efficiency ratio of 63.6%, delivering essential services like extractions, restorations, and preventive care to underserved areas [29]. Partnerships with the private sector to produce affordable preventive materials, such as fluoride toothpaste, have the potential to bridge resource gaps and likely support sustainable financing models [30, 31]. However, despite the potential of these approaches, studies on their scalability, integration, and long‐term impact in addressing oral health challenges in Africa remain limited. There is an urgent need for research to develop evidence‐based guidelines and roadmaps that build on theoretical and practical frameworks to optimize these strategies, ensuring equitable access and improved outcomes for African populations.

This scoping review aims to explore the current landscape of financing mechanisms for the prevention and control of NPDs in Africa. By mapping the available evidence, this review seeks to identify existing funding sources for the prevention and control of NPDs in Africa and the challenges associated with such funding sources, assess the effectiveness of existing financing models, and provide insights for policymakers and stakeholders to prioritize oral health within national health agendas. Ultimately, this review would assist decision‐makers in developing sustainable financing strategies that improve the oral health and overall well‐being of populations at risk of NPDs in Africa.

## 2 Methods

### 2.1 Title and Protocol Registration

The title and protocol of this scoping review has been registered in the Open Science Framework registry (https://osf.io/mqyt6).

### 2.2 Study Design

We utilized the design advanced by Arksey and O'Malley, which recommended a step‐by‐step approach for conducting scoping reviews [32]. Additionally, we adhered to the Preferred Reporting Items for Systematic Reviews and Meta‐analyses extension for Scoping Reviews guidelines (PRISMA‐ScR) in documenting this review [33].

### 2.3 Research Question

This review seeks to address this principal research question: How are the prevention and control of NPD financed in Africa? To robustly address this principal research question, it was further sub‐divided into the following sub‐questions:

i. What are the existing funding sources for NPD prevention and control in Africa?
ii. How effective are the current financing models for NPD prevention and control in Africa?
iii. What are the challenges associated with the financing of NPD prevention and control in Africa?

### 2.4 Definition of Key Terms

Necrotizing periodontal diseases have been classified based on the location of the tissue involved in the acute pathogenesis of the disease [33, 34]. These include:

- Necrotizing gingivitis: necrosis involving the gingival tissues.
- Necrotizing periodontitis: progression of necrosis into the periodontal ligament and the alveolar bone.
- Necrotizing stomatitis: progression of necrosis into the deeper tissues beyond the mucogingival line involving the tongue, cheek mucosa, or lip.

### 2.5 Review Selection Criteria

For this review, we utilized the following criteria to determine the eligibility of those publications that will be included in this scoping review:

- Articles published in peer‐reviewed journals.
- Articles published in English.
- Articles on empirical studies of any research design.
- Articles on empirical studies focusing on the financing of NPD prevention and control.
- Articles on empirical studies focusing on populations in African countries.
- Articles with accessible full texts.

Any publication that did not meet the above inclusion criteria were excluded from this review; these publications include grey literature, peer‐reviewed non‐original research articles (case reports, case series, systematic reviews, scoping reviews, meta‐analyses, and letters to the editor), articles published in French, Spanish, or any other non‐English language, articles with inaccessible full text (an article is considered inaccessible if it is not open access and it could not be obtained within 2 weeks after contacting the corresponding author), articles focusing on populations in European, Asian, American, Australian and Oceania countries, and articles focusing on the financing of health parameters other than NPDs.

### 2.6 Literature Search Strategy

We conducted a systematic search on April 1, 2025, to retrieve relevant studies from major bibliometric databases. Prior to this time, the research team met to deliberate and agree on the final approved search terms or keywords. Regarding the financing subject terms, we used the following terms: “money” or “grant*” or “fund*” or “sponsor*” or “financ*” or “loan” or “bank*” or “budget*” or “aid” or “donor” or “donat*” or “invest*” or “allocat*”.

The following terms were used for the NPD subject search: “Vincent's disease” or “Trench mouth” or “necroti*” or “stoma*” or “necroti*” or “periodont*” or “necroti*” or “gingiv*” or “noma” or “cancrum oris”.

Regarding African countries, territories, and dependencies subject search, the following terms were used: “Algeria” OR “Angola” OR “Benin” OR “Botswana” OR “burkina faso” OR “burundi” OR “cabo verde” OR “cape verde” OR “cameroon” OR “central african republic” OR “chad” OR “comoros” OR “congo” OR “ivory coast” OR “cote d ivoire” OR “djibouti” OR “democratic republic of congo” OR “egypt” OR “equatorial guinea” OR “eritrea” OR “eswatini” OR “ethiopia” OR “gabon” OR “gambia” OR “ghana” OR “guinea” OR “guinea bissau” OR “kenya” OR “lesotho” OR “liberia” OR “libya” OR “madagascar” OR “malawi” OR “mali” OR “mauritania” OR “mauritius” OR “morocco” OR “mozambique” OR “namibia” OR “niger” OR “nigeria” OR “rwanda” OR “sao tome and principe” OR “senegal” OR “seychelles” OR “sierra leone” OR “somalia” OR “south africa” OR “south sudan” OR “sudan” OR “tanzania” OR “togo” OR “tunisia” OR “uganda” OR “zambia” OR “zimbabwe” OR “reunion” OR “saint helena” OR “western sahara” OR “mayotte”. The search outcomes are presented in Supporting Information S1: Tables S1–S3.

Three authors (R.N., P.N., and K.K.K.) jointly conducted a systematic search using these agreed terms across the following databases: PubMed, SCOPUS, and AMED (These include the Allied and Complementary Medicine Database, CINAHL Ultimate, Dentistry and Oral Sciences Source, SPORTDiscus with Full Text, APA PsycArticles, Psychology and Behavioral Sciences Collection, and APA PsycInfo). See Supporting Informtion S1: Tables S1–S3 of the Supporting file for the search strings generated from the systematic search that we conducted.

### 2.7 Literature Selection

Citations of our search outcomes were retrieved from their associated databases, imported into the Rayyan software [35], and deduplicated. Based on the review's inclusion and exclusion criteria, two authors (R.A.N. and P.N.) independently and blindly performed title and abstract screening of all the retrieved records, while a third author (A.A.S.) resolved the conflicts that arose from the screening after extensively discussing with the other two authors (R.A.N. and P.N.). However, after the title and abstract screening phase, no literature record was considered for full text screening as none of them met the review's inclusion criteria. The outcomes of the study selection processes are presented in the results section of this article.

### 2.8 Quality Assessment of Included Literature

The authors intended to use the Mixed Methods Appraisal Tool (MMAT) 2018 version [36] to evaluate the quality of the studies included in this review. The MMAT was considered the most appropriate critical appraisal tool for this review because the tool is designed to evaluate the quality of empirical studies of diverse designs (qualitative, quantitative, and mixed methods study designs), which were anticipated to be included in this review. However, based on the literature selection outcome, quality assessment could not be done because no article was found eligible for inclusion into this review after thorough literature screening.

### 2.9 Data Charting

The following information were intended to be extracted for the review: author details, study title, country where study was conducted, study design, study sample size, type of NPD, sample characteristics (sex, age, and race/ethnicity), aim of study, method of participant recruitment or type of survey (household, hospital, clinic etc.), type of NPD financing (institutional government [federal, state, or local], institutional academic, non‐governmental organization, individual, faith‐based organization, and others), and the effectives and challenges of financing of NPD prevention and control in Africa. However, this could not be done because no article was found eligible for inclusion in this review after thorough literature screening.

### 2.10 Data Collation, Summarization, and Presentation

Collation, summarization, and presentation of the charted data were intended to be done in this scoping review using a thematic synthesis approach [37, 38]. However, this could not be accomplished because no article was found eligible for inclusion in this review after thorough literature screening.

### 2.11 Ethical Considerations

This study was a scoping review, and based on the scope of the study, no primary data were obtained from human or animal subjects. Hence, prior ethical clearance was not required to conduct this study.

## 3 Results

### 3.1 Literature Search Outcomes

**Figure 1:**
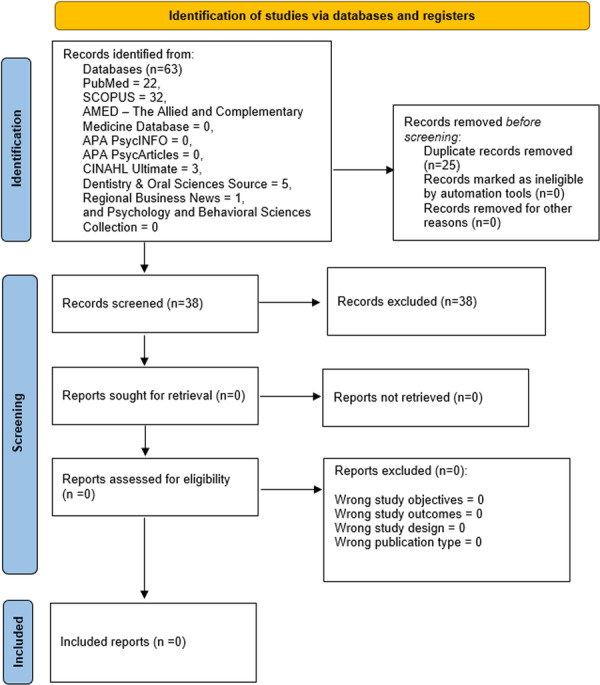
PRISMA 2020 flowchart detailing the systematic search and screening process of the scoping review, which resulted in the identification of no eligible studies for inclusion.

A comprehensive search across 10 databases yielded a total of 63 records (PubMed = 22; SCOPUS = 32; AMED—The Allied and Complementary Medicine Database = 0; CINAHL Ultimate = 3; Dentistry and Oral Sciences Source = 5; SPORTDiscus with Full Text = 0; APA PsycArticles = 0; Psychology and Behavioral Sciences Collection = 0; Regional Business News = 1; and APA PsycInfo = 0). Following the removal of 25 duplicate records on Rayyan, an initial screening process was conducted based on titles and abstracts of 38 unique studies. None of these met the predefined inclusion criteria. Therefore, no literature record was selected for full‐text review or subsequent analysis (Figure 1).

### 3.2 Review Findings

Being an empty scoping review, no relevant article was found to provide information on NPD financing in all the 54 countries, 2 dependencies, and 2 territories in Africa. Our review outcomes confirm that there is a significant gap in scientific evidence regarding the financing of NPD prevention and control in Africa.

## 4 Discussion

This scoping review aimed to identify existing funding sources and the associated challenges affecting the financing mechanisms for the prevention and control of NPD in Africa. After an extensive search across multiple databases and title and abstract screening, no studies met the inclusion criteria. This dearth of scientific evidence confirms a critical gap in the literature regarding the financial and economic aspects of NPD prevention and control in Africa. The disregard of NPD financing indicates general systemic gaps and the status of oral health within African health policies and global health funding.

Several contextual factors contribute to this gap. Notably, oral health remains a low priority within national health agendas and universal health coverage in Africa [13, 29, 39]. Regardless of its considerable burden and impacts on overall health and quality of life, merely 57% of countries on the continent have a national oral health policy [29]. In some countries, oral health is not incorporated into the general healthcare system as its training, service delivery, and financing, are distinct from the overall healthcare [40, 41]. This segregation affects systemic funding, integration of oral health into national healthcare financing frameworks, and access to oral healthcare services in Africa. In addition, limited research capacity is another contributory factor [29, 40].

Beyond this general prioritization challenge, the specific burden of NPDs is further overshadowed by competing demands from high‐profile diseases such as HIV/AIDS, tuberculosis, non‐communicable diseases, COVID‐19, cancer, and malaria. Due to limited resources, policymakers and donor agencies often allocate funding to conditions perceived as more imperative and matching the needs of low‐resource countries [42]. Even when oral health initiatives are funded, they tend to focus on certain oral conditions such as dental caries and oral cancer [43], while limited attention is given to financing periodontal disease prevention and control as shown by the results of this scoping review. Furthermore, in many African countries, oral diseases receive limited or no coverage under health insurance plans, with no government subsidies to offset costs [44, 45]. As a result, essential dental care services are inaccessible and unaffordable for populations already burdened by poverty and social determinants of health [8, 11].

Global health funding mechanisms further worsen these challenges. Determinants of funding priorities vary in different regions and are often influenced by donor's strategic objectives, values, and policy goals. Fund allocation is hardly determined by recipient country needs alone [46, 47]. Major funders like the Global Fund, Unitaid, Department for International Development, United States Agency for International Development, The Vaccine Alliance (GAVI), the Bill & Melinda Gates Foundation, and others prioritize diseases to fund based on global, political, economic, and technological agenda. Sometimes, contributors and partners, influence funding priorities and techniques of resource allocation [46]. Certain activities by some international funders, notably the use of advance market commitments (AMCs)—contracts by GAVI and provision of subsidies to vaccine manufacturers by the Bill & Melinda Gates Foundation, are driven by perceptions of profitability and alignment with global health investment frameworks [48]. Oral diseases, including NPD, may be seen as economically unappealing with limited returns on investment. This structural neglect worsens the chronic underfunding of oral health research and care, expanding disparities and contributing to avoidable morbidity and mortality.

The consequences of this neglect are profound. NPD, including necrotizing gingivitis and necrotizing periodontitis, imposes a significant and largely invisible oral health burden, especially among vulnerable Africans living with malnutrition and HIV/AIDS [8]. Malnutrition compromises immune responses, heightening susceptibility to severe periodontal infections, while immunocompromised HIV/AIDS patients experience accelerated disease progression and complications [49, 50]. Despite these well‐established interconnections between NPD and systemic conditions, financing for research, prevention, and care remains limited. This neglect results in late diagnoses, limited treatment options, and increased risk of disability and death, placing additional strain on already fragile healthcare systems across Africa [13].

One of the fundamental drivers of underfunding is the lack of comprehensive data on NPD prevalence, economic burden, and health impacts in Africa [11]. This data gap impedes advocacy [51] and could lead to an underestimation of the long‐term costs of poor NTD control. Addressing this requires prompt investment in health economic research to demonstrate the direct and indirect costs of NPD, including productivity losses and interactions with systemic diseases such as HIV/AIDS. Documenting the economic evidence will help inform cost‐effective, context‐appropriate strategies and financing mechanisms appropriate for African health systems.

Moving forward, sustainable and equitable financing models are essential. These must be contextualized to the realities of African healthcare systems and integrated into broader public health frameworks. Integrating NPD prevention and control into universal health coverage and existing disease programs can improve efficiency, access to care, and address enduring oral health inequities. Bridging the financing and knowledge gaps is critical for improving oral health outcomes, reducing NPD burden on individuals, families, communities, and healthcare systems, and consequently contributing to the global health and sustainable development goals.

## 5 Conclusion

In conclusion, this empty scoping review reveals a critical gap in the literature regarding the financing of NPD prevention and control in Africa. The absence of evidence underscores the urgent need for research and policy attention in this area. Context‐specific and sustainable financing strategies are essential to integrate NPD prevention into broader public health and universal health coverage frameworks. Addressing these gaps is vital for reducing oral health inequities and achieving global health and sustainable development goals. Future studies should explore innovative and equitable funding mechanisms tailored to low‐resource settings. Strengthening interdisciplinary collaboration among researchers, policymakers, and oral health professionals will be crucial to generate practical insights and support meaningful health system improvements.

## Author Contributions

**Kafayat Aminu:** conceptualization, investigation, writing – original draft, methodology, validation, visualization, writing – review and editing, software, formal analysis, project administration, data curation, supervision, resources. **Yovanthi Anurangi Jayasinghe:** writing – original draft, resources. **Oluwatobi Emmanuel Adegbile:** data curation, investigation, methodology, visualization, resources, validation, software, writing – original draft, writing – review and editing. **Adetayo Olorunlana:** writing – original draft, resources. **Rita Amarachi Nwebo:** conceptualization, data curation, formal analysis, investigation, methodology, visualization, project administration, resources, supervision, software, writing – original draft, writing – review and editing. **Precious Chika Nnannah:** conceptualization, investigation, writing – original draft, methodology, visualization, writing – review and editing, software, formal analysis, project administration, data curation, supervision, resources. **Afeez Abolarinwa Salami:** resources, investigation, methodology. **Akinyele Olumuyiwa Adisa:** resources, writing – review and editing. **Ugochukwu Anthony Eze:** resources, investigation. **Kehinde Kazeem Kanmodi:** conceptualization, data curation, funding acquisition, methodology, project administration, resources, software, supervision, validation, writing – original draft, writing – review and editing, investigation.

## Funding

The authors received no specific funding for this work.

## Ethics Statement

The authors have nothing to report.

## Conflicts of Interest

Kehinde Kazeem Kanmodi is an Editorial Board member of *Health Science Reports* and co‐author of this article. To minimize bias, he was excluded from all editorial decision‐making related to the acceptance of this article for publication. The other authors declare no conflicts of interest.

## Transparency Statement

The corresponding author, Kehinde Kazeem Kanmodi, affirms that this manuscript is an honest, accurate, and transparent account of the study being reported; that no important aspects of the study have been omitted; and that any discrepancies from the study as planned (and, if relevant, registered) have been explained.

## Supporting information


**Table S1:** Search string for PubMed database search. **Table S2:** Search string for SCOPUS database search. **Table S3:** Search string for other database (AMED – The Allied and Complementary Medicine Database, CINAHL Ultimate, Dentistry and Oral Sciences Source, SPORTDiscus with Full Text, APA PsycArticles, Psychology and Behavioral Sciences Collection, Regional Business News, and APA PsycInfo) search via EBSCO interface.

## Data Availability

The authors confirm that the data supporting the findings of this study are available within the article and its supporting materials.
