# Supplementary material for: An Empty Scoping Review on the Financing of Necrotizing Periodontal Disease Prevention and Control in Africa: Implications for Global Oral Health Research, Policy, and Practice
Source: Health Sci Rep. 2026 Feb 11;9(2):e71750. doi: 10.1002/hsr2.71750 (PMC12894775; doi:10.1002/hsr2.71750)
Supplement: Supplementary file 1 — Table S1: Search string for PubMed database search. Table S2: Search string for SCOPUS database search. Table S3: Search string for other database (AMED – The Allied and Complementary Medicine Database, CINAHL Ultimate, Dentistry and Oral Sciences Source, SPORTDiscus with Full Text, APA PsycArticles, Psychology and Behavioral Sciences Collection, Regional Business News, and APA PsycInfo) search via EBSCO interface. [file HSR2-9-e71750-s001.docx]

**SUPPLEMENTARY FILE**

**Table S1. Search string for PubMed database search**

| **Tag** | **Subject search** | **Search String** |
| --- | --- | --- |
| #1 | Financing | ((((((((((((money[Title/Abstract]) OR (grant*[Title/Abstract])) OR (fund*[Title/Abstract])) OR (sponsor*[Title/Abstract])) OR (financ*[Title/Abstract])) OR (loan[Title/Abstract])) OR (bank*[Title/Abstract])) OR (budget*[Title/Abstract])) OR (aid[Title/Abstract])) OR (donor[Title/Abstract])) OR (donat*[Title/Abstract])) OR (invest*[Title/Abstract])) OR (allocat*[Title/Abstract]) |
| #2 | Necrotizing Periodontal Disease | ((((((Vincent's disease[Title/Abstract]) OR (Trench mouth[Title/Abstract])) OR (necroti* stoma*[Title/Abstract])) OR (necroti* periodont*[Title/Abstract])) OR (necroti* gingiv*[Title/Abstract])) OR (noma[Title/Abstract])) OR (cancrum oris[Title/Abstract]) |
| #3 | African countries, territories, and dependencies | (((((((((((((((((((((((((((((((((((((((((((((((((((((((((((Algeria[Title/Abstract]) OR (Angola[Title/Abstract])) OR (Benin[Title/Abstract])) OR (Botswana[Title/Abstract])) OR (burkina faso[Title/Abstract])) OR (burundi[Title/Abstract])) OR (cabo verde[Title/Abstract])) OR (cape verde[Title/Abstract])) OR (cameroon[Title/Abstract])) OR (central african republic[Title/Abstract])) OR (chad[Title/Abstract])) OR (comoros[Title/Abstract])) OR (congo[Title/Abstract])) OR (ivory coast[Title/Abstract])) OR (cote d ivoire[Title/Abstract])) OR (djibouti[Title/Abstract])) OR (democratic republic of congo[Title/Abstract])) OR (egypt[Title/Abstract])) OR (equatorial guinea[Title/Abstract])) OR (eritrea[Title/Abstract])) OR (eswatini[Title/Abstract])) OR (ethiopia[Title/Abstract])) OR (gabon[Title/Abstract])) OR (gambia[Title/Abstract])) OR (ghana[Title/Abstract])) OR (guinea[Title/Abstract])) OR (guinea bissau[Title/Abstract])) OR (kenya[Title/Abstract])) OR (lesotho[Title/Abstract])) OR (liberia[Title/Abstract])) OR (libya[Title/Abstract])) OR (madagascar[Title/Abstract])) OR (malawi[Title/Abstract])) OR (mali[Title/Abstract])) OR (mauritania[Title/Abstract])) OR (mauritius[Title/Abstract])) OR (morocco[Title/Abstract])) OR (mozambique[Title/Abstract])) OR (namibia[Title/Abstract])) OR (niger[Title/Abstract])) OR (nigeria[Title/Abstract])) OR (rwanda[Title/Abstract])) OR (sao tome and principe[Title/Abstract])) OR (senegal[Title/Abstract])) OR (seychelles[Title/Abstract])) OR (sierra leone[Title/Abstract])) OR (somalia[Title/Abstract])) OR (south africa[Title/Abstract])) OR (south sudan[Title/Abstract])) OR (sudan[Title/Abstract])) OR (tanzania[Title/Abstract])) OR (togo[Title/Abstract])) OR (tunisia[Title/Abstract])) OR (uganda[Title/Abstract])) OR (zambia[Title/Abstract])) OR (zimbabwe[Title/Abstract])) OR (reunion[Title/Abstract])) OR (saint helena[Title/Abstract])) OR (western sahara[Title/Abstract])) OR (mayotte[Title/Abstract]) |
| #4 | #1 AND #2 AND #3 | ((#1) AND (#2)) AND (#3) |

**Table S2. Search string for SCOPUS database search**

| **Tag** | **Subject search** | **Search String** |
| --- | --- | --- |
| #1 | Financing | ( TITLE-ABS-KEY ( money ) OR TITLE-ABS-KEY ( grant* ) OR TITLE-ABS-KEY ( fund* ) OR TITLE-ABS-KEY ( sponsor* ) OR TITLE-ABS-KEY ( financ* ) OR TITLE-ABS-KEY ( loan ) OR TITLE-ABS-KEY ( bank* ) OR TITLE-ABS-KEY ( budget* ) OR TITLE-ABS-KEY ( aid ) OR TITLE-ABS-KEY ( donor ) OR TITLE-ABS-KEY ( donat* ) OR TITLE-ABS-KEY ( invest* ) OR TITLE-ABS-KEY ( allocat* ) ) |
| #2 | Necrotizing Periodontal Disease | ( TITLE-ABS-KEY ( "Vincent's disease" ) OR TITLE-ABS-KEY ( "Trench mouth" ) OR TITLE-ABS-KEY ( "necroti* stoma*" ) OR TITLE-ABS-KEY ( "necroti* periodont*" ) OR TITLE-ABS-KEY ( "necroti* gingiv*" ) OR TITLE-ABS-KEY ( noma ) OR TITLE-ABS-KEY ( "cancrum oris" ) ) |
| #3 | African countries, territories, and dependencies | ( ( TITLE-ABS-KEY ( angola )  OR  TITLE-ABS-KEY ( benin )  OR  TITLE-ABS-KEY ( botswana )  OR  TITLE-ABS-KEY ( "burkina faso" )  OR  TITLE-ABS-KEY ( burundi )  OR  TITLE-ABS-KEY ( cameroon )  OR  TITLE-ABS-KEY ( "cabo verde" )  OR  TITLE-ABS-KEY ( "cape verde" )  OR  TITLE-ABS-KEY ( "central african republic" )  OR  TITLE-ABS-KEY ( chad )  OR  TITLE-ABS-KEY ( comoros )  OR  TITLE-ABS-KEY ( congo )  OR  TITLE-ABS-KEY ( "ivory coast" )  OR  TITLE-ABS-KEY ( "democratic republic of congo" )  OR  TITLE-ABS-KEY ( djibouti )  OR  TITLE-ABS-KEY ( "equatorial guinea" )  OR  TITLE-ABS-KEY ( eritrea )  OR  TITLE-ABS-KEY ( ethiopia )  OR  TITLE-ABS-KEY ( gabon )  OR  TITLE-ABS-KEY ( gambia )  OR  TITLE-ABS-KEY ( ghana )  OR  TITLE-ABS-KEY ( guinea )  OR  TITLE-ABS-KEY ( guinea-bissau )  OR  TITLE-ABS-KEY ( kenya )  OR  TITLE-ABS-KEY ( lesotho )  OR  TITLE-ABS-KEY ( liberia )  OR  TITLE-ABS-KEY ( madagascar )  OR  TITLE-ABS-KEY ( malawi )  OR  TITLE-ABS-KEY ( mali )  OR  TITLE-ABS-KEY ( mauritania )  OR  TITLE-ABS-KEY ( mauritius )  OR  TITLE-ABS-KEY ( mayotte )  OR  TITLE-ABS-KEY ( mozambique )  OR  TITLE-ABS-KEY ( namibia )  OR  TITLE-ABS-KEY ( niger )  OR  TITLE-ABS-KEY ( nigeria )  OR  TITLE-ABS-KEY ( reunion )  OR  TITLE-ABS-KEY ( rwanda )  OR  TITLE-ABS-KEY ( "saint helena" )  OR  TITLE-ABS-KEY ( sao  AND tome  AND  principe )  OR  TITLE-ABS-KEY ( senegal )  OR  TITLE-ABS-KEY ( seychelles )  OR  TITLE-ABS-KEY ( "sierra leone" )  OR  TITLE-ABS-KEY ( somalia )  OR  TITLE-ABS-KEY ( "south africa" )  OR  TITLE-ABS-KEY ( "south sudan" ) ) )  OR  ( ( TITLE-ABS-KEY ( eswatini )  OR  TITLE-ABS-KEY ( togo )  OR  TITLE-ABS-KEY ( uganda )  OR  TITLE-ABS-KEY ( zambia )  OR  TITLE-ABS-KEY ( zimbabwe )  OR  TITLE-ABS-KEY ( egypt )  OR  TITLE-ABS-KEY ( libya )  OR  TITLE-ABS-KEY ( algeria )  OR  TITLE-ABS-KEY ( tunisia )  OR  TITLE-ABS-KEY ( morocco )  OR  TITLE-ABS-KEY ( "western sahara" )  OR  TITLE-ABS-KEY ( sudan )  OR  TITLE-ABS-KEY ( tunisia ) ) ) |
| #4 | #1 AND #2 AND #3 | ( ( TITLE-ABS-KEY ( "Vincent's disease" ) OR TITLE-ABS-KEY ( "Trench mouth" ) OR TITLE-ABS-KEY ( "necroti* stoma*" ) OR TITLE-ABS-KEY ( "necroti* periodont*" ) OR TITLE-ABS-KEY ( "necroti* gingiv*" ) OR TITLE-ABS-KEY ( noma ) OR TITLE-ABS-KEY ( "cancrum oris" ) ) ) AND ( ( TITLE-ABS-KEY ( money ) OR TITLE-ABS-KEY ( grant* ) OR TITLE-ABS-KEY ( fund* ) OR TITLE-ABS-KEY ( sponsor* ) OR TITLE-ABS-KEY ( financ* ) OR TITLE-ABS-KEY ( loan ) OR TITLE-ABS-KEY ( bank* ) OR TITLE-ABS-KEY ( budget* ) OR TITLE-ABS-KEY ( aid ) OR TITLE-ABS-KEY ( donor ) OR TITLE-ABS-KEY ( donat* ) OR TITLE-ABS-KEY ( invest* ) OR TITLE-ABS-KEY ( allocat* ) ) ) AND ( ( ( TITLE-ABS-KEY ( angola ) OR TITLE-ABS-KEY ( benin ) OR TITLE-ABS-KEY ( botswana ) OR TITLE-ABS-KEY ( "burkina faso" ) OR TITLE-ABS-KEY ( burundi ) OR TITLE-ABS-KEY ( cameroon ) OR TITLE-ABS-KEY ( "cabo verde" ) OR TITLE-ABS-KEY ( "cape verde" ) OR TITLE-ABS-KEY ( "central african republic" ) OR TITLE-ABS-KEY ( chad ) OR TITLE-ABS-KEY ( comoros ) OR TITLE-ABS-KEY ( congo ) OR TITLE-ABS-KEY ( "ivory coast" ) OR TITLE-ABS-KEY ( "democratic republic of congo" ) OR TITLE-ABS-KEY ( djibouti ) OR TITLE-ABS-KEY ( "equatorial guinea" ) OR TITLE-ABS-KEY ( eritrea ) OR TITLE-ABS-KEY ( ethiopia ) OR TITLE-ABS-KEY ( gabon ) OR TITLE-ABS-KEY ( gambia ) OR TITLE-ABS-KEY ( ghana ) OR TITLE-ABS-KEY ( guinea ) OR TITLE-ABS-KEY ( guinea-bissau ) OR TITLE-ABS-KEY ( kenya ) OR TITLE-ABS-KEY ( lesotho ) OR TITLE-ABS-KEY ( liberia ) OR TITLE-ABS-KEY ( madagascar ) OR TITLE-ABS-KEY ( malawi ) OR TITLE-ABS-KEY ( mali ) OR TITLE-ABS-KEY ( mauritania ) OR TITLE-ABS-KEY ( mauritius ) OR TITLE-ABS-KEY ( mayotte ) OR TITLE-ABS-KEY ( mozambique ) OR TITLE-ABS-KEY ( namibia ) OR TITLE-ABS-KEY ( niger ) OR TITLE-ABS-KEY ( nigeria ) OR TITLE-ABS-KEY ( reunion ) OR TITLE-ABS-KEY ( rwanda ) OR TITLE-ABS-KEY ( "saint helena" ) OR TITLE-ABS-KEY ( sao AND tome AND principe ) OR TITLE-ABS-KEY ( senegal ) OR TITLE-ABS-KEY ( seychelles ) OR TITLE-ABS-KEY ( "sierra leone" ) OR TITLE-ABS-KEY ( somalia ) OR TITLE-ABS-KEY ( "south africa" ) OR TITLE-ABS-KEY ( "south sudan" ) ) ) OR ( ( TITLE-ABS-KEY ( eswatini ) OR TITLE-ABS-KEY ( togo ) OR TITLE-ABS-KEY ( uganda ) OR TITLE-ABS-KEY ( zambia ) OR TITLE-ABS-KEY ( zimbabwe ) OR TITLE-ABS-KEY ( egypt ) OR TITLE-ABS-KEY ( libya ) OR TITLE-ABS-KEY ( algeria ) OR TITLE-ABS-KEY ( tunisia ) OR TITLE-ABS-KEY ( morocco ) OR TITLE-ABS-KEY ( "western sahara" ) OR TITLE-ABS-KEY ( sudan ) OR TITLE-ABS-KEY ( tunisia ) ) ) ) |

**Table S3. Search string for other database (AMED – The Allied and Complementary Medicine Database, CINAHL Ultimate, Dentistry and Oral Sciences Source, SPORTDiscus with Full Text, APA PsycArticles, Psychology and Behavioral Sciences Collection, Regional Business News, and APA PsycInfo) search via EBSCO interface**

| **Tag** | **Subject search** | **Search String** |
| --- | --- | --- |
| S1 | Financing | AB money OR AB grant* OR AB fund* OR AB sponsor* OR AB financ* OR AB loan OR AB bank* OR AB budget* OR AB aid OR AB donor OR AB donat* OR AB invest* OR AB allocat* |
| S2 | Necrotizing Periodontal Disease | AB Vincent’s disease OR AB Trench mouth OR AB necroti* stoma* OR AB necroti* periodont* OR AB necroti* gingiv* OR AB noma OR AB cancrum oris |
| S3 | African countries, territories, and dependencies | AB algeria OR AB angola OR AB benin OR AB botswana OR AB burkina faso OR AB burundi OR AB cape verde OR AB cabo verde OR AB cameroon OR AB central african republic OR AB chad OR AB comoros OR AB congo OR AB cote d'ivoire OR AB ivory coast OR AB djibouti OR AB democratic republic of congo OR AB egypt OR AB equatorial guinea OR AB eritrea OR AB eswatini OR AB ethiopia OR AB gabon OR AB gambia OR AB ghana OR AB guinea OR AB guinea bissau OR AB kenya OR AB lesotho OR AB liberia OR AB libya OR AB madagascar OR AB malawi OR AB mali OR AB mauritania OR AB mauritius OR AB morocco OR AB mozambique OR AB namibia OR AB niger OR AB nigeria OR AB rwanda OR AB ( sao tome and principe ) OR AB senegal OR AB seychelles OR AB sierra leone OR AB somalia OR AB south Africa OR AB south sudan OR AB sudan OR AB tanzania OR AB togo OR AB tunisia OR AB uganda OR AB zambia OR AB zimbabwe OR AB reunion OR AB saint helena OR AB western sahara OR AB mayotte |
| S4 | S1 AND S2 AND S3 | S1 AND S2 AND S3 |
